# Australian Pregnant Women's Awareness of Gestational Weight Gain and Dietary Guidelines: Opportunity for Action

**DOI:** 10.1155/2016/8162645

**Published:** 2016-01-06

**Authors:** Khlood Bookari, Heather Yeatman, Moira Williamson

**Affiliations:** ^1^School of Health and Society, Faculty of Social Sciences, University of Wollongong, Wollongong, NSW 2522, Australia; ^2^School of Nursing, University of Wollongong, Wollongong, NSW 2522, Australia; ^3^School of Nursing and Midwifery, CQ University, Noosaville, QLD 4566, Australia

## Abstract

*Background*. Excessive gestational weight gain (GWG) can negatively impact on maternal and foetal health. Guidelines based on Institute of Medicine (IOM) encourage managing GWG by following healthy eating recommendations and increasing physical activity. This study investigated pregnant women's knowledge of their optimal GWG and recommended dietary approaches for GWG management.* Method*. English-speaking pregnant women were recruited from five hospitals in New South Wales (Australia) and an online link. Prepregnancy Body Mass Index (BMI) was calculated from self-reported height and prepregnancy weight. Participants identified their recommended GWG. A survey assessed practical dietary knowledge and asked about broad dietary recommendations to prevent excessive GWG. Chi square and logistic regression analyses were used.* Results*. *N* = 326 pregnant women completed the surveys; 49% entered pregnancy overweight (25.2%) or obese (23.6%); and knowledge of recommended GWG was lacking. Prepregnancy BMI was a significant predictor of GWG recommendation knowledge (*P* < 0.000). Pregnant women were highly knowledgeable about broad dietary recommendations but had poor knowledge of detailed recommendations.* Conclusions*. Limited knowledge of IOM's GWG guidelines and of specific dietary recommendations for pregnancy should be addressed by health care providers and education initiatives to assist the high number of women who enter pregnancy overweight or obese.

## 1. Introduction

Excessive gestational weight gain (GWG) is prevalent in obstetric populations worldwide [1–3]. Excessive GWG has the potential to negatively impact on maternal and foetal health, causing a range of poor obstetric and neonatal outcomes. Adverse obstetric outcomes include increased risk of maternal complications such as gestational diabetes [4], postpartum weight retention [5], and development of long-term obesity [6]. Foetal exposure to maternal obesity, diabetes, and excessive GWG can increase their risk of childhood obesity [3] and chronic diseases later in life [7].

The Institute of Medicine (IOM) in the United States (US) published revised evidence-based guidelines for appropriate GWG [3] which have been adopted by other developed countries including Australia [8, 9]. The revised guidelines provide recommendations for appropriate GWG according to prenatal BMI categories. According to the IOM's report on GWG, ideal weight gain, nutrition, and physical activity are important elements in both pregnancy outcome and the long-term health of mother and child [3].

Knowledge alone is not enough to change behaviours and bring about positive outcomes, but it is considered to be a necessary prerequisite [10]. A recent Australian study found that pregnant women's views of their expected weight gain were a significant predictor of their actual gestational weight gain (GWG) [11]. A meta-analysis of studies that aimed at improving GWG and pregnancy outcomes through dietary and lifestyle interventions also found that dietary interventions which included dietary advice were associated with the largest reduction in women's GWG and resulted in some improvement in pregnancy outcomes compared with other lifestyle change interventions (physical activity alone, and a mixed approach which combined diet and physical activity) [12].

While all pregnant women should aim to gain weight within the recommended weight range, restricted dietary regimes are not recommended [8]. To combat excessive GWG, minimising the intake of energy in combination with consuming adequate amounts of nutrient dense foods from each of the five food groups and increasing physical activity are recommended [8]. However, many pregnant women do not comply with these recommendations and poor nutrition knowledge could be a contributing factor [13, 14]. Pregnant women have been reported to consume high amounts of processed, energy-dense, salty foods and sugary drinks [2]. Regular consumption of such foods in pregnancy is an established risk factor for excessive GWG and obesity.

With the increasing availability of energy-dense and nutrient-poor foods that are high in sugar and fat [15], Worsley et. al. (2014) argued that public awareness of the presence of high levels of fat, sugar, and salt in such foods is “more important than ever” [16]. Thus it is wise for women to be aware of the existence of these nutrients in foods so they can make mindful food choices. In addition, increased portion sizes of many foods have the potential to be translated into extra energy intake [17] which can contribute to extra weight gain. Increased awareness by pregnant women (especially those who enter pregnancy overweight or obese) about portion size has been identified as a beneficial tool that can be used in the context of GWG management [18]. It has been found that 3.7% of the variability in GWG was uniquely explained by portion size factor [18].

Official dietary guidelines provide evidence-based information about which foods a pregnant woman should eat, how much a serve is, and how many serves a day of particular foods should be consumed to achieve a healthy diet and to achieve the recommended GWG [19]. However, little has been reported on Australian women's knowledge of these guidelines in the context of GWG management. Such information may play a key role in characterizing women's awareness about safe dietary approaches for weight gain management during pregnancy.

Earlier studies have investigated either women's awareness in relation to recommendations of Australian Guidelines for Healthy Eating (AGHE) [20, 21] excluding GWG guidelines or pregnant women's knowledge of the GWG guidelines and a broader range of dietary approaches to attain effective GWG management [22]. However, studies have indicated that individuals tend to have better knowledge of the broad dietary guidelines (e.g., eat more of fruit and vegetables and less fat) and poor knowledge about specific topics of nutrition (e.g., nutrient content of foods and applying knowledge to food choices) [14, 23]. Thus research is required that focuses on both the general and detailed components of nutrition knowledge that women can apply in daily life to achieve a healthy and balanced diet according to AGHE for pregnant woman.

The aim of the study was to assess (1) pregnant women's accuracy of estimating their GWG according to IOM guidelines and (2) pregnant women's awareness of the broad dietary recommendations and detailed dietary approaches necessary to achieve a healthy diet and better GWG management. The demographic characteristics of pregnant women associated with their level of knowledge were also investigated.

## 2. Methods

### 2.1. Survey Development

The survey instrument was derived from a multidimensional survey that assessed women's dietary adherence to AGHE, attitude, motivation for dietary change and knowledge of AGHE during pregnancy, and other recommendations for staying healthy and active during pregnancy, including the IOM guidelines for GWG and its management. The survey also included demographic questions on women's education, stage of pregnancy, age, marital status, income, language, and self-reported prepregnancy BMI. The multidimensional survey included 95 items and was developed using existing [24] and validated surveys [16, 23, 25] and newly created questions appropriate to the purpose of this study.

The current analysis focused on pregnant women's attitudes and confusion about and knowledge of guidelines for GWG and its management, including relevant aspects of women's nutritional knowledge.

To explore women's attitudes toward the importance of knowing about recommended GWG participants were asked to select from a five-point Likert scale: not important, somewhat important, important, very important, and essential. Likewise for the misunderstanding about recommended GWG, participants were asked to select from a five-point Likert scale: not at all confused, somewhat confused, confused, very confused, and do not understand at all. Participants were asked if they were aware of the weight they were recommended to gain over the course of their pregnancy and to specify it in kilograms. Answers were compared with the 2009 IOM guidelines for their prepregnancy BMI category and coded into three classes: an “underestimate,” “correct,” or “overestimate” (resp., a figure less than, within, or greater than the guidelines). They also indicated whether their weight had been monitored during pregnancy.

To assess participants' knowledge about broad approaches for GWG management, participants were asked to indicate whether a number of behaviours (dietary and exercise related) helped prevent excessive GWG. Participants' knowledge about the energy requirements during pregnancy was also assessed.

Participants were asked to indicate whether they were aware of AGHE for pregnancy (this response was not included in the scoring). Thirty-six questions assessed some detailed practical aspects of the participant's dietary knowledge, including knowledge of dietary recommendations for major food groups (5 items), food proportions and standard serving sizes of selected food items (14 items), selecting most energy-dense macronutrient (1 item), the presence of nutrients (sugar, fat, and dietary fibre) in selected food products (13 items) that may impact GWG, and choosing everyday foods (3 items). Answers were weighted as correct = 1 point and incorrect answers and the “not sure” option = 0.

The survey was pilot tested with four dietitians, a statistician, ten pregnant women, and five researchers at the University of Wollongong (UOW) and amended accordingly. The study was approved by the University of UOW Human Research and Ethics committee (HREC) as well as the South East Sydney Illawarra Area Health Services, South Western Sydney Local Health District Sites (Campbelltown/Liverpool hospitals), utilising the National Ethic Application Form (NEAF) [26]. The survey was designed to be administered online and self-completed and participation was anonymous.

### 2.2. Survey Administration

Pregnant women were invited to participate via public pregnancy/baby expositions (Wollongong); in free sample/information bags distributed to pregnant women at New South Wales (NSW) hospitals/pharmacies; at two NSW baby retail stores; and in antenatal clinic waiting rooms in five NSW hospitals. Women attending expositions and antenatal clinics were provided with verbal information on the survey's purpose and informed that participation was voluntary. If they agreed to participate they were provided with an iPad to complete the survey online at the time or an information leaflet with a link to complete the survey online at a later time. These recruitment methods were implemented between October 2012 and July 2013. Additional participants were recruited via a snowball effect whereby survey participants promoted the survey to friends verbally and through social media. Participants were eligible to enter a draw for three 50-dollar gift vouchers if they completed the survey. The study was limited to English-speaking pregnant women.

### 2.3. Statistical Analysis

Women's self-reported prepregnancy BMI was calculated and participants were classified according to WHO BMI classification [27]. Raw data were downloaded from the SurveyMonkey website and the iPads and transferred to the Statistical Package of the Social Sciences software (Version 22.0. Armonk, NY: IBM Corp). Chi square and Fisher's exact tests were used to assess the associations between demographic factors and participants' accuracy to estimate the recommended GWG for their pregnancy. A logistic regression was performed to ascertain the effects of independent factors on the likelihood that participants overestimated their recommended GWG. Significant differences were identified at *P* < 0.05.

## 3. Results

### 3.1. Study Sample Characteristics

Responses were received from 472 pregnant women. Analysis included only the results from the 326 participants who fully completed all survey's sections and provided information allowing calculation of their BMI (72 did not complete the demographic section and 74 did not provide either prepregnancy self-reported height or weight). Only 46.6% of participants were classified as being normal weight (BMI 18.5–24.99 kg/m^2^); nearly 49% were either “overweight” (25.2%; BMI 25–29.9 kg/m^2^) or obese (23.6%; BMI ≥ 30 kg/m^2^). Of participants classified as obese, 45.5% (*n* = 35) were classified as obese class 1 (BMI 30.0–34.9 kg/m^2^), 32.5% (*n* = 25) as obese class 2 (BMI 35.0–39.9 kg/m^2^), and 22% (*n* = 17) as obese class 3 (BMI ≥ 40.0 kg/m^2^). Almost two-thirds (66.3%, *n* = 216) of the participants reported that their weight had been monitored. Of the 326 participants, 79.8% (*n* = 260) were from NSW, with the remainder (20.2%; *n* = 66) from elsewhere in Australia. More than half (56%) held a university degree (Table 1).

### 3.2. Women's GWG Related Attitude and Confusion

More than half of participants indicated that knowing about recommended GWG was “very important” (35.6%; *n* = 116) or “essential” (20.6%; *n* = 67). Less than one-third (31%; *n* = 101) considered it as “important,” 11.3% (*n* = 37) as “somewhat important,” and only 1.5% (*n* = 5) as “not important.” The majority of participants indicated that they were “not at all confused” (60.1%; *n* = 196) or “somewhat confused” (30.4%; *n* = 99) about GWG recommendations. Very few indicated they were “confused” (6.1%; *n* = 20), “very confused” (1.8%; *n* = 6), or “do not understand at all” (1.5%; *n* = 5) about the recommended GWG topic.

### 3.3. Women's Awareness of GWG Recommendations

Although 63.2% (*n* = 206) indicated that they were aware of the recommended GWG for their pregnancy, only 27.6% (*n* = 57) of them correctly indicated the recommended GWG. Overweight and obese women were least accurate in estimating the appropriate GWG (*P* < 0.000). More than half of overweight women (62.2%) and of obese women (55.5%) overestimated the recommended GWG for their own pregnancy (Table 2).

### 3.4. Broad Knowledge about Energy, Dietary, and Exercise Recommendations

A four-item question sought to assess participants' knowledge of recommended dietary approaches and exercise for the prevention of excessive GWG (Table 3). Most participants were able to recite the correct answers about the broad nutrition recommendations that can help them prevent excessive GWG. Most of the correct answers pertained to following dietary approach options: eating “less dietary fibre” was not a way to reduce GWG (81.3%, *n* = 265); eating “less saturated fat” (88.7%, *n* = 289) and eating “more fruit and vegetables” (89.3%  *n* = 291) were ways to reduce GWG. The majority of the participants (76.7%, *n* = 250) were also able to recognise the plate that represented a healthy dinner plate. Likewise, more than three-quarters of the participants chose the correct answers in all 3 questions on energy requirements. Inversely, about eight out of ten women (82.8%) inaccurately indicated that engaging in at least 30 minutes of vigorous exercise on daily basis could help prevent excessive GWG.

### 3.5. Detailed Dietary Recommendations

Nearly two-thirds (63.8%, *n* = 208) of participants stated they were not familiar with the AGHE for pregnant women. Although 36.2% (*n* = 118) of participants indicated that they were familiar with the AGHE, no difference was found in this group's knowledge of recommended number of daily serves of the five food groups compared to those who indicated they were not aware of the AGHE (*P* < 0.63). Less than half of participants were aware of the recommended number of serves for fruit and vegetables (46.6%, *n* = 152), bread and cereals (34.4%, *n* = 112), and protein food groups (28.8%, *n* = 94). Participants' abilities to identify standard serving sizes of food items varied with the type of food, ranging from 11% (yoghurt) to 81% (medium potato).

Participants' awareness of macronutrient composition of common foods and energy density of fat was assessed. Lowest scores were evident for dietary fibre content of cornflakes with only 56.1% (*n* = 183) identifying it as a low source of fibre, and saturated fat content of avocado with only 60.7% (*n* = 198) of participants identifying it as a low source of saturated fat. Around 77.3% of participants (*n* = 252) were unable to recognise that fat had more kilojoules (i.e., calories, energy) than sugar and alcohol for the same weight of product.

Factors found to have a statistically significant (*P* < 0.05) association with the dependent variable using Crosstab Chi square analyses for group variables were controlled in the final model. Only participants' number of children (*P* < 0.033) and women's prepregnancy BMI (*P* < 0.000) were significantly associated with the accuracy of estimation of GWG recommendations. A logistic regression was performed to ascertain the effects of these former factors on the likelihood of pregnant women to overestimate their recommended GWG. The logistic regression model was statistically significant, *χ* ^2^ (2, *N* = 108) = 34.041, *P* < 0.0005. The model explained 36.1% (Nagelkerke *R* ^2^) of the variance in women's accuracy of estimation of GWG recommendations and correctly classified 73% of cases. About 91% of participants who overestimated their GWG were also predicted by the model to overestimate the recommended GWG range. While 56% of participants who did correctly estimate their GWG were correctly predicted by the model to identify the recommended GWG range. Positive predictive value (out of all cases predicted as overestimated, the percentage correctly predicted as overestimated) was 67% and negative predictive value (out of all the cases of correctly estimating their GWG, the percentage correctly predicted as correct) was 86%. Of the two predictor variables only women's prepregnancy BMI was significant. Compared with normal weight participants, obese and overweight had 12 and 13 times (95% CI for odds ratios: 4–38; 95% CI for odds ratios: 4–46) higher odds, respectively, to overestimate the recommended GWG (*P* < 0.000), controlling for the other factor in the model.

## 4. Discussion

This study assessed Australian pregnant women's knowledge relating to GWG. The results confirm a notable lack of maternal knowledge about guidelines of appropriate GWG and the practical details of dietary guidelines (e.g., how many serves are recommended a day? How large is each serve?). Of particular note was that prepregnancy BMI was found to have a strong association with women's accuracy to estimate the recommended GWG.

The participants demonstrated misconceptions about their recommended GWG as prescribed by the 2009 IOM guidelines, even though 87.2% had indicated that knowing about recommended GWG was important, very important, or essential to them. The vast majority reported they were not confused about GWG recommendations and more than 63% were aware of the recommended GWG but only a few participants (*n* = 57) correctly identified the recommended range. This finding of pregnant women's limited knowledge of appropriate GWG is consistent with previous studies [1, 14, 28]. Lack of knowledge about appropriate GWG may arise if pregnant women do not have or seek access to accurate information about the GWG recommendations. This may particularly be the case as the majority of participants (erroneously) believed they were already aware of such information. This may help to explain the high rate of noncompliance with the GWG recommendations.

Overweight and obese women were less accurate in identifying the recommended GWG than their normal weight peers, with 62.2% of overweight and 55.5% of obese pregnant women overestimating their recommended GWG, which is similar to studies in Canada [29] and earlier Australian studies [1, 22, 30]. In this study women's prepregnancy BMI was found to be a strong predictor for accuracy in estimating the recommended GWG. Women who entered pregnancy as being overweight and obese were substantially (12 and 13 times) more likely to report recommended GWG above the IOM recommendations than those who were within the healthy weight range. This level of misreporting is in line with previous research [31]. Such findings are unsurprising, as previous studies have found that inadequate or inappropriate advice is given to pregnant women, with the majority of overweight/obese women either being advised to gain too much weight or not receiving any advice for weight gain during pregnancy from their caregivers [1, 30–32]. Maternal reported target weight gain is one of the factors that is potentially modifiable and has been shown to be strongly associated with actual gestational weight gain [11, 33]. In light of our findings, women entering pregnancy at risk due to heavier weight are additionally at high risk of exceeding their recommended GWG.

In our study, although the majority of women showed a high level of knowledge of the broad dietary recommendations, their awareness of the detailed messages was poor. For example, women were aware of the general key dietary approaches to manage excessive gestational weight gain by reducing the intake of added sugar and fat and increasing the intake of dietary fibre. However, lack of knowledge was evident for the more detailed questions about how many serves there are a day, how much a serve is, and the presence, type, and energy density of fat. These results are in line with earlier studies [14, 21, 23]. This suggests nutrition promotion campaigns about broadly based dietary approaches have effectively reached the public including pregnant women and have been successful in increasing their awareness. However detailed information essential in making mindful food choices is still limited. This situation has the potential to make the management of excessive GWG even more challenging.

Despite the importance of adhering to the dietary and GWG guidelines, women's knowledge about these topics remains poor. This may reflect the lack of communication about these topics between women and their health care providers. It has been reported that more attention is paid to informing women about foods to avoid (e.g., those potentially containing* Listeria monocytogenes*) whilst topics like GWG and adequate fruit and vegetables remain a low priority for health care providers [34, 35]. Healthcare providers have been reported to focus more on topics that they think have obvious, immediate, and severe impacts on pregnancy and are less likely to consider long-term public health initiatives. Although* Listeria* has obvious, immediate, and severe impacts on pregnancy (e.g., miscarriages and stillbirth), the accumulated risks inherent in poor nutrition practices and excessive GWG would be far greater, as it is increasingly common and causes a range of poor obstetric and neonatal outcomes. A narrow view of risk limits the provision of accurate and succinct information about dietary guidelines from trusted sources of information for pregnant women. These results indicate that a change of focus is warranted.

Exercise and being active are considered key factors for healthy pregnancy in women of all weight ranges. The current guidelines recommend pregnant women engage in moderate-intensity exercise for 30 minutes a day on most, if not all, days of the week [36]. Our results revealed that most pregnant women (82.8%) incorrectly associated engaging in vigorous exercise as one of the ways that could help in managing weight gain in pregnancy. This misconception may reflect the fact that exercise is not generally included when health professionals discuss GWG with pregnant women [32]. Pregnant women have been reported to be less active than nonpregnant women, with activity decreasing over pregnancy [37]. Thus encouraging pregnant women to undertake moderate rather than vigorous activity might be the first step to attain recommended GWG, as it is more based on women's normal activity levels [38].

Previous studies have generated contradictory results regarding the value of frequent weighing in reducing excess GWG [39, 40]. About 66.3% of the pregnant women in our study reported that their weight had been monitored but they did not indicate by whom. Routine measurement of maternal weight according to recommended guidelines may be important in the context of enabling caregivers to better track women's GWG, to schedule regular opportunities to communicate the dietary and physical activity advice that pregnant women are currently lacking, and to provide support for women who are at risk of losing weight or of excessive GWG [34]. 

*Limitations and Strength of the Study*. Due to the nonrepresentative nature of the convenience sampling, the results of this study cannot be generalised. Cultural differences in women's knowledge about the nutrition and GWG recommendations were not measured and commented on as maternal ethnicity was not included in this study and it was administered in English, thus excluding women for whom English was not their main language. Self-reported weight and height were used to calculate the BMI which may be inaccurate; however, it has been reported that self-reported weight and height are positively correlated with the measured anthropometrics [41].

The strength of this study is that the results provide additional valuable insights into the gap in GWG and nutrition knowledge in a large sample of English-speaking Australian pregnant women. This information can be used to inform interventions with this group. Deepening the knowledge of factors that can assist in promoting favourable dietary behaviours may support targeted interventions to prevent excess GWG and future weight gain in women and their offspring.

The present study reveals that pregnant women were aware of the general dietary guidelines but did not know the detailed and practical side of them. Questions outside the scope of current study that need to be addressed in future studies include the following: “To what extent can women apply their current knowledge about the general dietary guidance in practice?”; “do dietary practices pose any harm (in terms of insufficient intake or overconsumption) to their babies and their own health?”; and “what time, type, level, and format of information are needed to initiate healthy eating behaviours and better assist women in preventing excessive GWG?”

## 5. Conclusion

The findings of this study shed light on pregnant women's level of knowledge about GWG, and physical and dietary recommendations. These findings reinforce the importance for health care providers who see pregnant women on regular basis to give targeted, accurate, and practical advice to all pregnant women. Further it highlights the particular needs of overweight and obese pregnant women for targeted attention to the recommended guidelines for healthy weight gain during pregnancy. As women's behaviours have been found to be influenced by their caregivers' guidance about GWG [42, 43], all health professionals who provide care for pregnant women should be prepared to counsel women on the benefits of gaining weight within the appropriate range during their pregnancy and to inform them about IOM's guidelines.

## Figures and Tables

**Table 1:** Characteristics of the study sample.

Characteristics	Entire sample
	Total *n* = 326 (%)
*Prior pregnancies*	
None	169 (51.8%)
One	107 (32.8%)
Two and more	50 (15.3%)
*Stage of pregnancy*	
First trimester	35 (10.7%)
Second trimester	126 (38.7%)
Third trimester	165 (50.6%)
*Planned pregnancy*	
Yes	271 (83.1%)
No	55 (16.9%)
*Age*	
Under 20 years	7 (2.1%)
20–29 years	162 (49.7%)
30–39 years	146 (44.8%)
40 years and above	11 (3.4%)
*Marital status*	
Single	13 (4%)
Married/de facto	310 (95.1%)
Separated/divorced/widowed	3 (.9%)
*Education*	
Some high school or less	20 (6.1%)
High school completed	42 (12.9%)
TAFE	79 (24.2%)
Tertiary education	185 (56.7%)
*Household income*	
Less than $25,000/yr	33 (10.1%)
$25,000–$50,000/yr	73 (22.4%)
More than 50,000/yr	220 (67.5%)
*First language*	
English	273 (83.7%)
Other	53 (16.3%)
*Having health and nutrition related qualification*	
Yes	61 (18.7%)
No	265 (81.3%)
*Seen by dietitian and/or nutritionist*	
Yes	96 (29.4%)
No	230 (70.6%)
*Prepregnancy BMI*	
Underweight	15 (4.6%)
Normal	152 (46.6%)
Overweight	82 (25.2%)
Obese	77 (23.6%)
*Are you aware of recommended GWG for your pregnancy?*	
Yes	206 (63.2%)
No	120 (36.8%)

Data in *n* (%).

**Table 2:** Women's accuracy in estimating their recommended GWG according to their prepregnancy BMI category.

	Total number^*∗*^	Estimated recommended weight gain			
		Underestimate	Correct	Overestimate	
Women classified according to BMI category					
Normal	83	47 (56.6%)	31 (37.3%)	5 (6%)	*P* < 0.000
Overweight	45	2 (4.4%)	15 (33.3%)	28 (62.2%)	
Obese	36	7 (19.4%)	9 (25%)	20 (55.5%)	

Data in *n* (%)

^*∗*^Total number of women who provided an estimation of their recommended gestational weight gain (GWG).

**Table 3:** Women's awareness of the recommended approaches to achieve healthy diet and better GWG management.

			Correct

Broad nutrition knowledge		*Do you think this behaviour help in preventing too much weight gain during pregnancy*	
		Eating less dietary fibre	265 (81.3%)
		Eating less saturated fat	289 (88.7%)
		Eating more fruit and vegetables	291 (89.3%)
		Engaging in at least 30 minutes of vigorous exercise every day	56 (17.2)
		*Energy requirements in pregnancy*	
		Do you think women's energy requirements (kilojoules/day) change during pregnancy?	303 (92.9%)
		Do you think pregnant women's energy requirements (kilojoules/day) varies according to the different stages (trimesters) of pregnancy?	293 (89.9%)
		Do you think there is a need to increase food quantity during pregnancy (“eating for two adults”) for the benefit of the baby?	252 (77.3%)

Detailed nutrition knowledge	How many serves are recommended a day?	*What do you think is the recommended number of servings of*:	
		Fruit and vegetables	152 (46.6%)
		Dairy foods	181 (55.5%)
		Protein foods	94 (28.8%)
		Bread and cereals	112 (34.4%)
		Extra foods	288 (88.3%)
		
	How much is a serve?	*Do the following amounts of foods represent one serve, less than one or more than one serve*:	
		2 slices of bread	210 (64.4%)
		1.5 cup of breakfast cereal flakes or porridge	177 (54.3%)
		3/4 cup cooked rice, pasta and noodles	61 (18.7)
		1 medium potato	264 (81%)
		5 strawberries	90 (27.6%)
		20 grapes	151 (46.3%)
		150 g yoghurt	36 (11%)
		20 g (1 slice) cheese	124 (38%)
		100 g of meat & chicken	248 (76.1%)
		80–120 g cooked fish fillet	254 (77.9%)
		1 chocolate bar	190 (58.3%)
		1 scoop of ice cream	47 (14.4%)
		1 meat pie	225 (69%)
		*A healthy dinner plate should consist of 1/2 vegetables, 1/4 protein and 1/4 carbohydrates*	250 (76.7%)
		
	Nutrients content of common food items	*Food source is high/low of following nutrients*	
		Sugar	
		Bananas	275 (84.4%)
		Strawberry yoghurt	230 (70.6%)
		Orange 35% juice	295 (90.5%)
		Muesli bar	264 (81%)
		Dietary fibre	
		Cornflakes	183 (56.1%)
		Bananas	245 (75.2%)
		Wholegrain bread	307 (94.2%)
		Fish	219 (67.2%)
		Saturated fat	
		Lean red meat	266 (81.6%)
		Whole milk	211 (64.7%)
		Avocado	198 (60.7%)
		Vegetarian pastry	210 (64.4%)
		*Which of these breads contains the most dietary fibre? *(a)* white bread, *(b)* wholemeal bread, *(c)* wholegrain bread *	231 (70.9%)
		*Select the most energy dense macronutrient*	74 (22.7%)
		
	Food choices	*The best choice for a low fat, high fibre light meal*	250 (76.7%)
		*The healthier option of spaghetti bolognese (proportion of carbohydrates to fat)*	48 (14.7%)
		*The healthier option of sweet but low in add sugar food*	280 (85.9%)

Data in *n* (%).

## Abbreviations

AGHE: Australian Guide for Healthy Eating
BMI: Body Mass Index
GWG: Gestational weight gain
IOM: The Institute of Medicine
NSW: New South Wales
SPSS: Statistical Package of the Social Software
SD: Standard deviation
UOW: University of Wollongong.
