# Corrigendum to “Australian Pregnant Women's Awareness of Gestational Weight Gain and Dietary Guidelines: Opportunity for Action”

**DOI:** 10.1155/2017/9372040

**Published:** 2017-07-13

**Authors:** Khlood Bookari, Heather Yeatman, Moira Williamson

**Affiliations:** ^1^School of Health and Society, Faculty of Social Sciences, University of Wollongong, Wollongong, NSW 2522, Australia; ^2^School of Nursing, University of Wollongong, Wollongong, NSW 2522, Australia; ^3^School of Nursing and Midwifery, CQ University, Noosaville, QLD 4566, Australia

In the article titled “Australian Pregnant Women's Awareness of Gestational Weight Gain and Dietary Guidelines: Opportunity for Action” [1], there was an error and missing reference in the first paragraph of Section “2.1. Survey Development,” where [2] should be added to the article as [44] and cited in the following statement “The survey instrument was derived from a multidimensional survey that assessed women's dietary adherence to AGHE, attitude, motivation for dietary change and knowledge of AGHE during pregnancy, and other recommendations for staying healthy and active during pregnancy, including the IOM guidelines for GWG and its management [44].” Meanwhile, the number of survey items should be 109 instead of 95 in the statement “The survey also included demographic questions on women's education, stage of pregnancy, age, marital status, income, language, and self-reported prepregnancy BMI. The multidimensional survey included 95 items and was developed using existing [24] and validated surveys [16, 23, 25] and newly created questions appropriate to the purpose of this study.”

Additionally, [36] was incomplete. It should be corrected as “36. Australian Health Ministers' Advisory Council. ‘Clinical practice guidelines: Antenatal care—module 2,' Australian Government Department of Health and Ageing: Canberra, 2014.”
